# Synthesis of Thioethers by InI_3_-Catalyzed Substitution of Siloxy Group Using Thiosilanes

**DOI:** 10.3390/molecules21101330

**Published:** 2016-10-06

**Authors:** Yoshihiro Nishimoto, Aya Okita, Akio Baba, Makoto Yasuda

**Affiliations:** 1 Frontier Research Base for Global Young Researchers, Center for Open Innovation Research and Education (COiRE), Graduate School of Engineering, Osaka University, Osaka 565-0871, Japan; 2 Department of Applied Chemistry, Graduate School of Engineering, Osaka University, Osaka 565-0871, Japan; ayaokita@chem.eng.osaka-u.ac.jp (A.O.); baba@chem.eng.osaka-u.ac.jp (A.B.)

**Keywords:** indium, silyl ethers, thiosilanes, thioethers

## Abstract

The substitution of a siloxy group using thiosilanes smoothly occurred in the presence of InI_3_ catalyst to yield the corresponding thioethers. InI_3_ was a specifically effective catalyst in this reaction system, while other typical Lewis acids such as BF_3_·OEt_2_, AlCl_3_, and TiCl_4_ were ineffective. Various silyl ethers such as primary alkyl, secondary alkyl, tertiary alkyl, allylic, benzylic, and propargylic types were applicable. In addition, bulky OSi*t*BuMe_2_ and OSi*i*Pr_3_ groups, other than the OSiMe_3_ group, were successfully substituted. The substitution reaction of enantiopure secondary benzylic silyl ether yielded the corresponding racemic thioether product, which suggested that the reaction of tertiary alkyl, secondary alkyl, benzylic, and propargylic silyl ethers would proceed via a S_N_1 mechanism.

## 1. Introduction

**Scheme 1:**
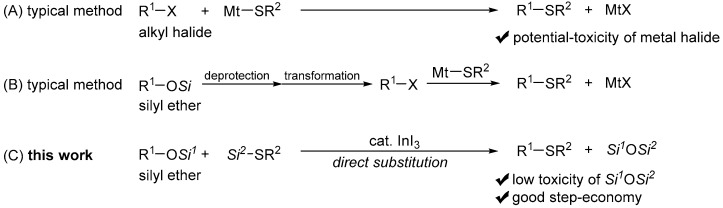
Synthetic methods for producing a thioether. (Mt = metal, R^1^ = alkyl, R^2^ = alkyl or aryl).

Organosulfur compounds are important building blocks in organic synthesis because many natural and pharmaceutical products contain sulfur [1,2,3,4,5]. In particular, a thioether is a popular and useful compound [1,2,3,4,5]. Therefore, there are various types of synthetic methods to produce thioethers such as hydrothiolation of alkenes [6,7,8,9], Chan-Lam-Evans coupling using thiols [10], and transition metal-catalyzed coupling between aryl halides and thiols [11,12]. The substitution reaction of alkyl halides with sulfur nucleophiles is one of the most typical and practical methods in the synthesis of alkyl thioethers (Scheme 1A) [13,14,15,16,17,18]. However, the use of alkyl halides has an inherent problem; that is, the potential toxicity of alkyl halides and metal halides as by-products. Recently, alcohol derivatives such as alkyl ethers, alkyl acetates, alkyl carbonates, and silyl ethers have been suggested as promising substrates that could solve the problem. In particular, silyl ethers are one of the most useful alcohol derivatives because they are often used as protected alcohols in the syntheses of complex organic compounds such as natural products, drugs, and agrichemicals [19,20]. However, there are few reports about the synthesis of thioethers via the direct use of silyl ethers, due to the very poor leaving ability of the siloxy group [21]. Although the coupling reaction between alkenyl silyl ethers and thiosilanes has been reported, a stoichiometric amount of BF_3_·OEt_2_ was required [22]. Electrolysis with a thiosilane using only an α-acylamino silyl ether was also reported [23]. Therefore, in general, a multi-step sequence involving deprotection and transformation of the siloxy group is required in order to transform silyl ethers to thioethers (Scheme 1B). Therefore, the establishment of a direct transformation of silyl ethers to thioethers would be ideal in terms of step-economy. Herein, we report the direct substitution of a siloxy group with thiosilanes catalyzed by InI_3_ to synthesize a variety of thioethers (Scheme 1C). A disiloxane generated as a by-product has low toxicity, is inert, and is easily removed, so the present substitution reaction is a very practical synthetic method for producing thioethers.

## 2. Results

First, the effect of the catalyst was investigated in the reaction of the primary alkyl silyl ether **1a** with trimethyl(phenylthio)silane (**2a**) (Table 1). Recently, we studied the moderate Lewis acidity of indium salts in order to develop the catalytic coupling reactions of alcohols and their derivatives with various nucleophiles [24,25,26,27,28,29,30,31,32,33,34]. Therefore, a substitution reaction in the presence of InI_3_ at room temperature was carried out and produced the desired thioether **3aa** with a 27% yield (Entry 1). The InI_3_-catalyzed reaction at 80 °C moderately proceeded to produce **3aa** with a 53% yield (Entry 2). By contrast, InCl_3_ did not mediate this substitution reaction (Entry 3), and InBr_3_ gave only a 27% yield (Entry 4). Typical Lewis acids such as BF_3_·OEt_2_, AlCl_3_, and TiCl_4_ showed no catalytic activity (Entries 5–7). The use of nonpolar solvents such as toluene and hexane resulted in low yields (Entries 8 and 9). Polar tetrahydrofuran (THF) solvent was not suitable (Entry 10). Finally, the InI_3_-catalyzed reaction carried out in ClCH_2_CH_2_Cl at 80 °C for 8 h produced the highest yield (Entry 11).

The scope of the silyl ethers is listed in Table 2. The reaction of secondary alkyl silyl ether **1b** resulted in only a 32% yield of thioether **3ba** (Entry 1). On the other hand, tertiary alkyl and secondary benzyl silyl ethers (**1c** and **1d**) gave very high yields even at room temperature (Entries 2 and 3). Primary benzylic substrates were also suitable for this system, and both electron-rich and electron-poor benzyl silyl ethers produced the corresponding thioethers **3ea**, **3fa**, and **3ga** in high yields (Entries 4–6). The substitution reaction of propargylic silyl ether **1h** smoothly occurred at room temperature without an allenylic thioether product being generated in a rearrangement reaction (Entry 7). Additionally, the primary alkyl silyl ether **1i**, which bears an olefin moiety, was applicable to this reaction (Entry 8).

**Scheme 2:**
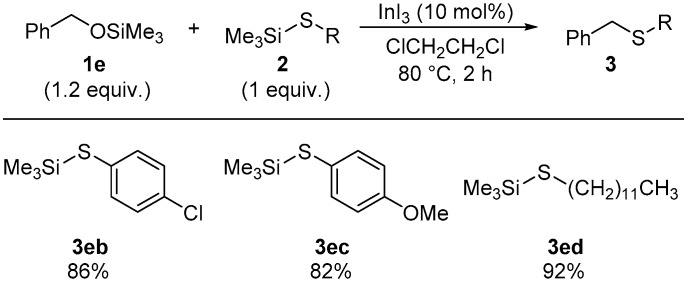
Substitution reaction using different types of thiosilanes.

Various types of thiosilanes were examined in this reaction system (Scheme 2). Arylthiosilanes bearing electron-withdrawing and electron-donating groups produced the desired products **3eb** and **3ec** in high yields, respectively. An alkyl thiosilane, other than an aryl type, was also applicable to the present substitution reaction. The reaction of benzyl silyl ether **1e** with trimethyl(dodecylthio)silane (**2d**) smoothly occurred to produce the corresponding dialkyl thioether **3ed** with 92% yield.

**Scheme 3:**
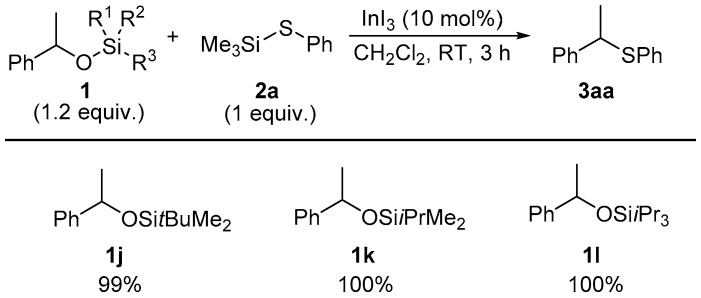
Substitution of bulky siloxy groups. RT: room temperature.

Bulky silyl groups are generally more useful and robust protecting groups compared with the trimethylsilyl group in organic synthesis. We examined OSi*t*BuMe_2_, OSi*i*PrMe_2_, and OSi*i*Pr_3_ groups for the substitution reaction (Scheme 3). Despite the large steric hindrance, the bulky silyl ethers **1j**, **1k**, and **1l** reacted with thiosilane **2a** to produce the corresponding thioether **3aa** in high yields.

**Scheme 4:**
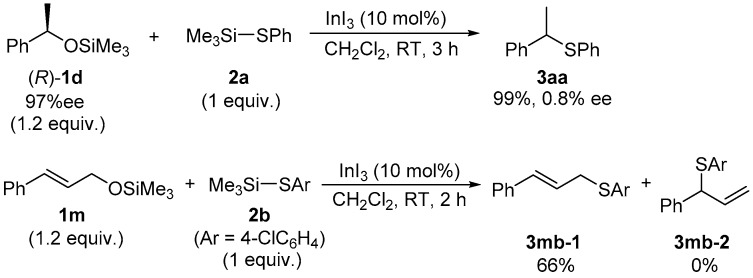
Mechanistic study.

The excellent results given by the reaction using tertiary alkyl and benzylic thioethers suggested that the substitution reaction using these thioethers occurred via the S_N_1 mechanism involving a carbocation intermediate. Actually, the reaction of the enantiopure benzyl silyl ether (*R*)-**1d** catalyzed by InI_3_ produced a racemic mixture of **3aa** (Scheme 4, upper line) [35]. The reaction using the allyl silyl ether **1m** exclusively yielded thioether **3mb-1** without producing the thioether **3mb-2** through allylic rearrangement (S_N_2′ mechanism) (Scheme 4, lower line). This result showed that the reaction of a primary allylic silyl ether involves a S_N_2 mechanism.

**Scheme 5:**
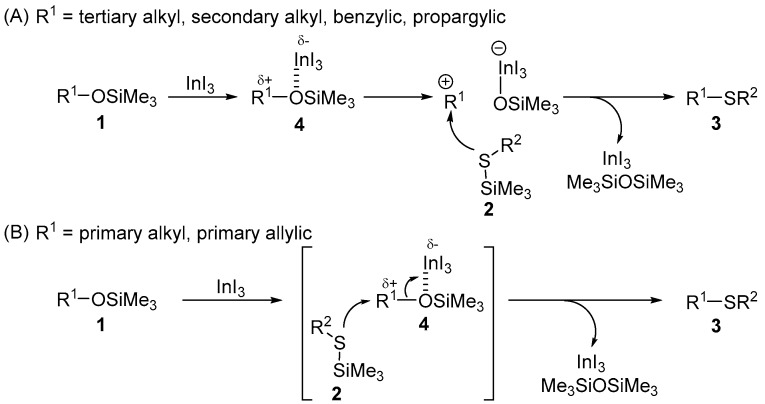
Plausible reaction mechanisms.

Plausible reaction mechanisms are illustrated in Scheme 5. From the result of Equation 1, the substitution reactions of tertiary alkyl, secondary alkyl, benzylic, and propargylic silyl ethers would proceed via the S_N_1 mechanism (Scheme 5A). A siloxy group coordinates to InI_3_ (**4**), and then the cleavage of the C–O bond generates a carbocation intermediate. The nucleophilic attack of the thiosilane **2** to the carbocation intermediate gives thioether **3** and Me_3_SiOSiMe_3_, and InI_3_ regenerates. On the other hand, the reaction of a primary alkyl silyl ether would proceed via a S_N_2-type mechanism (Scheme 5B), because a primary alkyl cation is not easily generated. First, the coordination of a siloxy group to InI_3_ enhances polarization of the C–O bond. Then, an S_N_2 reaction of the InI_3_-activated silyl ether **4** with thiosilane **2** occurs. The reaction of a primary allyl silyl ether also involves this type of mechanism (Scheme 4, lower line). Generally, transmetalation between a metal salt (MtX_n_) and thiosilane (R^2^S-SiMe_3_) may occur to generate a metal thiolate (MtX_n−y_(SR^2^)_y_). Actually, AlCl_3_ and BF_3_·OEt_2_ transmetalate with thiosilane **2a** to form thioaluminum and thioborane, respectively [36]. On the other hand, the transmetalation between InI_3_ and thiosilane **2a** does not occur, which allows InI_3_ to work as a Lewis acid catalyst in the present substitution reaction [36]. A disiloxane by-product has low toxicity and is easily removed by column chromatography on silica gel, which enhances the utility of this reaction system in organic synthesis.

## 3. Experimental Section

Typical Procedure: Silyl ether **1c** (0.135 g, 0.6 mmol) was added to a suspended solution of thiosilane **2a** (0.089 g, 0.5 mmol) and InI_3_ (0.026 g, 0.05 mmol) in dichloromethane (0.5 mL). The reaction mixture was stirred at room temperature for 2 h and was then quenched by a saturated aqueous solution of NaHCO_3_. The crude product was extracted with dichloromethane. The combined organic layer was dried over MgSO_4_, and concentrated under reduced pressure. The NMR yield was determined by ^1^H-NMR (^1^H-NMR spectra were recorded on a JMTC-400/54/SS instrument at 400 MHz (JEOL Ltd., Tokyo, Japan), using 1,1,2,2-tetrachloroethane as an internal standard. The crude product was purified by flash chromatography (Hexane/EtOAc = 95:5, spherical silica gel 60 μm, 30 g, diameter 2.7 cm, Shoko Scientific Co., Ltd., Kanagawa, Japan) to afford the corresponding thioether **3ca** (0.119 g, 95%).

## 4. Conclusions 

We have developed an InI_3_-catalyzed coupling reaction of silyl ethers with thiosilanes. A variety of silyl ethers and thiosilanes are applicable to the present coupling reaction. In particular, the scope of silyl ethers is significantly broad, and primary alkyl, secondary alkyl, tertiary alkyl, benzylic, and propargylic silyl ethers are feasible substrates. In addition, the substitution of OSiMe_3_ as well as OSi*t*BuMe_2_ and OSi*i*Pr_3_ groups smoothly occurred. InI_3_ specifically achieved this catalytic substitution reaction unlike other typical Lewis acids. This was possible because the transmetalation between InI_3_ and thiosilane does not occur, and InI_3_ sufficiently activates silyl ether due to its moderate Lewis acidity.

## Figures and Tables

**Table 1:**
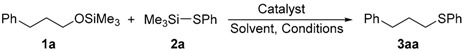
Optimization of conditions in the reaction of silyl ether **1a** with thiosilane **2a** ^a^.

Entry	Catalyst (10 mol %)	Solvent	Conditions	Yield (%) ^b^
1	InI_3_	CH_2_Cl_2_	RT ^c^, 2 h	27
2	InI_3_	ClCH_2_CH_2_Cl	80 °C, 2 h	53
3	InCl_3_	ClCH_2_CH_2_Cl	80 °C, 2 h	0
4	InBr_3_	ClCH_2_CH_2_Cl	80 °C, 2 h	27
5	BF_3_·OEt_2_	ClCH_2_CH_2_Cl	80 °C, 2 h	0
6	AlCl_3_	ClCH_2_CH_2_Cl	80 °C, 2 h	0
7	TiCl_4_	ClCH_2_CH_2_Cl	80 °C, 2 h	0
8	InI_3_	Toluene	80 °C, 2 h	30
9	InI_3_	Hexane	68 °C, 2 h	17
10	InI_3_	THF ^d^	66 °C, 2 h	0
11	InI_3_	ClCH_2_CH_2_Cl	80 °C, 8 h	67

^a^ **1a** (1.2 equiv.), **2a** (1 equiv.), catalyst (0.1 equiv.), solvent (1 M); ^b^ Yields were determined by ^1^H-NMR; ^c^ RT = room temperature; ^d^ THF = Tetrahydrofuran.

**Table 2:**
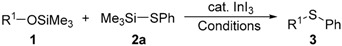
Scope of the silyl ethers **1b–i** in the InI_3_-catalyzed substitution reaction using thiosilane **2a** ^a^.

Entry	R^1^OSiMe_3_	Conditions	Product	Yield (%) ^b^
1	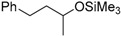 **1b**	ClCH_2_CH_2_Cl 80 °C, 8 h	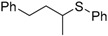 **3ba**	32
2	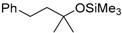 **1c**	CH_2_Cl_2_ RT, 2 h	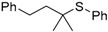 **3ca**	99 (95) ^c^
3	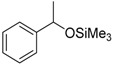 **1d**	CH_2_Cl_2_ RT, 2 h	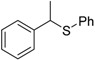 **3da**	98
4	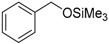 **1e**	ClCH_2_CH_2_Cl 80 °C, 2 h	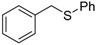 **3ea**	85
5	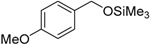 **1f**	CH_2_Cl_2_ RT, 2 h	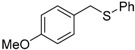 **3fa**	88
6	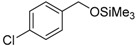 **1g**	ClCH_2_CH_2_Cl 80 °C, 2 h	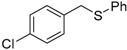 **3ga**	83
7	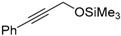 **1h**	CH_2_Cl_2_ RT, 2 h	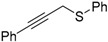 **3ha**	67
8	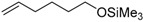 **1i**	ClCH_2_CH_2_Cl 80 °C, 2 h	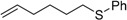 **3ia**	36

^a^ **1** (1.2 equiv.), **2a** (1 equiv.), InI_3_ (0.1 equiv.), solvent (1 M); ^b^ Yields were determined by ^1^H-NMR; ^c^ Isolated yield.
